# Exploration of the breast ductal carcinoma in situ signature and its prognostic implications

**DOI:** 10.1002/cam4.5071

**Published:** 2022-07-26

**Authors:** Jiao Zhang, Hui Lin, Lei Hou, Hui Xiao, Xilong Gong, Xuhui Guo, Xuchen Cao, Zhenzhen Liu

**Affiliations:** ^1^ Department of Breast Disease, Henan Breast Cancer Center Affiliated Cancer Hospital of Zhengzhou University & Henan Cancer Hospital Zhengzhou China; ^2^ The First Department of Breast Cancer Tianjin Medical University Cancer Institute and Hospital, National Clinical Research Center for Cancer, Key Laboratory of Cancer Prevention and Therapy, Tianjin's Clinical Research Center for Cancer, Key Laboratory of Breast Cancer Prevention and Therapy, Tianjin Medical University, Ministry of Education Tianjin China; ^3^ Taizhou Hospital of Zhejiang Province Affiliated to Wenzhou Medical University Taizhou China

**Keywords:** biomarker, ductal carcinoma in situ, gene signature, prognosis

## Abstract

Following the implementation of breast screening programs, the occurrence of ductal carcinoma in situ (DCIS) as an early type of neoplasia has increased. Although the prognosis is promising, 20%–50% of DCIS patients will progress to invasive ductal carcinoma (IDC) if not treated. It is essential to look for promising biomarkers for predicting DCIS prognosis. The Gene Expression Omnibus (GEO) database was used to explore the expression of genes that differed between DCIS and normal tissue in this investigation. Enrichment analysis was performed to characterize the biological role and intrinsic process pathway. The Cancer Genome Atlas Breast Cancer Dataset was used to categorize the hub genes, and the results were confirmed using the Cytoscape plugin CytoHubba and MCODE. The prognostic ability of the core gene signature was determined through time‐dependent receiver operating characteristic (ROC), Kaplan–Meier survival curve, Oncomine databases, and UALCAN databases. In addition, the prognostic value of core genes was verified in proliferation assays. We identified 217 common differentially expressed genes (DEGs) in the present study, with 101 upregulated and 138 downregulated genes. The top genes were obtained from the PPI network (protein–protein interaction). A unique six‐gene signature (containing GAPDH, CDH2, BIRC5, NEK2, IDH2, and MELK) was developed for DCIS prognostic prediction. Centered on the Cancer Genome Atlas (TCGA) cohort, the ROC curve showed strong results in prognosis prediction. The six core gene signatures is often overexpressed in DCIS, with a weak prognosis. Furthermore, when breast cancer cells are transfected with small interfering RNAs, downregulation of core gene expression substantially inhibits cell proliferation, revealing a high potential for employing core genes in DCIS prognosis. In conclusion, the current investigation verified the six core genes signatures for prospective DCIS biomarkers, which may aid clinical decision‐making for individual care.

## Graphical abstract

Following the implementation of breast screening programs, the occurrence of Ductal carcinoma in situ (DCIS) as an early type of neoplasia has increased.The current investigation verified the six core genes (containing GAPDH, CDH2, BIRC5, NEK2, IDH2, and MELK) signatures for prospective DCIS biomarkers, which may aid clinical decision‐making for individual care.

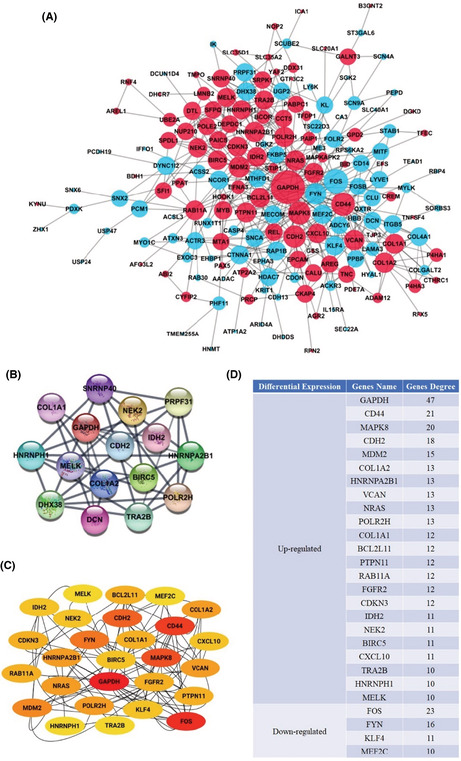

## 1 INTRODUCTION

According to the GLOBOCAN 2020 profile produced by the International Agency for Research on Cancer, breast cancer has overtaken lung cancer as the world's most commonly diagnosed cancer, with estimated new cases of 2.3 million.^1^ With the advancement of drug discovery, clinical trials, and the evolution of treatment concepts, the 5‐year survival rate for breast cancer has risen to over 90%. However, recurrence and metastasis remain the leading causes of breast cancer therapy failure.^2^, ^3^ As a result, early detection and treatment have become critical procedures for improving survival and reducing patient suffering.

Ductal carcinoma in situ (DCIS) is a proliferation of neoplastic luminal cells restricted to the duct lobular system of the breast and is also considered an early stage of cancer. Patients with DCIS who have substantial calcification on mammography but no symptoms are frequently diagnosed.^4^ Following the implementation of breast screening programs, the prevalence of DCIS has risen, accounting for 20%–25% of all breast cancer cases.^5^, ^6^ Depending on the hormone receptor expression status, traditional treatment involves mastectomy or breast‐conserving surgery, radiotherapy, and, in some cases, endocrine treatment.^7^, ^8^ Long‐term outcomes of DCIS after management show high local control rates with excellent overall survival.^9^, ^10^, ^11^ If left untreated, DCIS is expected to advance to invasive ductal carcinoma (IDC) in 20%–50% of cases. It is still challenging to predict which lesions will progress to IDC and which will not. The specific biomarkers for DCIS diagnosis and prognosis in clinical practice are undetermined.^12^, ^13^, ^14^

As genome‐sequencing technology has advanced, evidence has emerged that differentially expressed genes hold tremendous potential in diagnosis and prognosis of DCIS. A gene microarray profile can be used to uncover novel biomarkers that can help with diagnosis and individualized treatment. Hence, the present study aims to conduct a microarray profile dataset analysis obtained through the gene expression omnibus (GEO) database, perform an integrated DCIS analysis, and identify potential biomarkers. The Cancer Genome Atlas Breast Cancer Dataset will be used to confirm the expression of these genes, and enrichment analysis will be utilized to clarify the biological role and intrinsic mechanism pathway. Additionally, in proliferation assays, the prognostic value of core genes will be verified.

## 2 MATERIALS AND METHODS

### 2.1 Gene expression microarray data

We used the GSE7882,^15^ GSE21422,^16^ and GSE59246^4^ microarray profile datasets acquired from the Gene Expression Omnibus (GEO, https://www.ncbi.nlm.nih.gov/geo/) for this study. The GSE7882 is based on the GPL5326 platform (NCI Qiagen Homo sapiens 36 K v3 cgh expression), including 93 DCIS tissues and 7 benign epithelium tissues. The GSE21422 is based on the GPL570 platform (Affymetrix Human Genome U133 Plus 2.0 Array), including nine DCIS samples and five healthy breast tissue samples. While, the GSE59246 is based on the GPL13607 platform (Agilent‐028004 SurePrint G3 Human GE 8x60K Microarray), which includes 46 DCIS samples and 3 noncancerous breast tissues.

### 2.2 DEGs identification

Differentially expressed genes (DEGs) between DCIS samples and noncancerous tissues were determined through GEO2R (https://www.ncbi.nlm.nih.gov/geo/geo2r/). The GEO2R can compare two or more sample groups using the analysis of variance or the *t‐*test as an R programming language‐based dataset analysis tool in the GEO series datasets. Adjusted *p*‐value <0.05 and |log FC| > 1 were set as the cutoff criteria. BioDBnet (https://biodbnet‐abcc.ncifcrf.gov/db/db2db.php) database was utilized to convert identifiers from Gene ID to Gene symbol. DEGs were used to evaluate the overlapping genes in the three microarray profile datasets using FunRich software (version 3.1.3).

### 2.3 Pathway enrichment analysis and gene ontology

The Database for Annotation, Visualization, and Integrated Discovery (DAVID, version 6.8, https://david.ncifcrf.gov/) and FunRich Software (version 3.1.3) was utilized to determine the biological role of candidate DEGs and possible pathway enrichment. *p*‐value <0.05 is the cutoff criterion for pathway analysis and significant function.

### 2.4 PPI network construction and hub gene identification

To predict the candidate DEG protein–protein interaction (PPI) network, we used the Search Tool for the Retrieval of Interacting Genes database (STRING, version 11.0, http://string‐db.org), with 0.400 medium confidence and confidence network edges as the product criterion. The PPI network was then built and analyzed the candidate DEG encoding protein interactions through the Cytoscape software (version 3.7.2, http://www.cytoscape.org/). CytoHubba and MCODE, Cytoscape two plugins, were then utilized to explore the hub genes of the PPI network, and the node degree was calculated, which is the number of interconnections to filter PPI hub genes.

### 2.5 Validation of the identified hub genes

Based on the Cancer Genome Atlas (TCGA) database, the Metabolic gEne RApid Visualizer (MERAV, http://merav.wi.mit.edu/) was utilized for the expression validation of hub genes. The MERAV website is really for analyzing human gene expression through a variety of arrays. Primary tumors, normal tissues, and cancer and non‐cancer cell line arrays were normalized together to generate a gene expression database that provides a means of consistent comparison.

### 2.6 Hub gene signatures and prognosis analysis

Hub gene expression was evaluated by UALCAN (http://ualcan.path.uab. edu/index.html) in molecular subtypes and nodal metastasis. The importance of the prognosis of the known hub genes was examined through the Kaplan–Meier plotter (https://kmplot.com/analysis/), an online database capable of evaluating the impact of genes on survival in various cancer types. The analysis was limited to specified cohorts with estrogen receptor positive or negative, progesterone receptor positive or negative, HER2 positive or negative, and lymph node negative.

### 2.7 Cell culture

The Chinese Academy of Sciences Cell Bank (Shanghai, China) provided the T47D, MCF10A, SK‐BR‐3, MDA‐MB‐231, MCF‐7, BT549, and BT474 cell lines. MCF10A was cultured in DMEM (Dulbecco's modified Eagle's medium) 5% horse serum (Gibco) supplemented with F12. T47D and MCF‐7 cells were grown in 10% fetal bovine serum (FBS) supplemented with DMEM. SK‐BR‐3, BT549, and BT474 were maintained in 10% FBS supplemented with RPMI‐1640 medium. Cell culture of MDA‐MB‐231 cell lines was prepared in 10% FBS supplemented with Leibovitz's L‐15 medium in the presence of CO_2_ at 37°C.

### 2.8 RNA extraction and reverse transcription‐quantitative polymerase chain reaction (RT‐qPCR)

Total RNA was extracted from cultured cells using Trizol reagent (Takara). The SuperScript RT kit (Takara) was utilized for reverse transcription. The SYBR Green PCR package (Takara) was used for the RT‐qPCR assay. Table S1 contains the PCR primer sequences used in this study. The relative expression levels were calculated through the 2^−ΔΔCt^ method, where the Ct values represent the average of each gene in triplicate reactions.

### 2.9 Transfection and small interfering RNA (siRNA)

siRNAs of hub genes and the appropriate scrambled control were acquired from RiboBio. siRNA target sequences are listed in Table S2. According to the supplier's instructions, the transfection of siRNAs with different cell lines was performed using FuGENE HD Transfection Reagent (Promega).

### 2.10 Proliferation assays

Cell proliferation potential was assessed using MTT, colony formation, and EdU assays. MTT assay was conducted by seeding 2 × 10^3^ cells in 96‐well plates after the transfection of 24 h. Twenty microliters of MTT (0.5 mg/ml, Solarbio) was added to the cells after indicated time, then incubated for 4 h at 37°C, followed by the medium removal and the Formosan precipitate solubilization in 150 μl of DMSO. A microplate reader was used to test the activities of the viable cells at 570 nm. At the same time, 8 × 10^2^ cells were seeded for 2 weeks in 6‐well plates in the colony formation assay. The colonies were fixed for 30 min in 4% paraformaldehyde before being stained with hematoxylin and counted and compared to a control group. According to the manufacturer's instructions, the EdU test was performed using the Cell‐Light Edu Apollo488 In Vitro Imaging Kit (RiboBio). EdU‐positive cell percentage was calculated under the fluorescence microscope.

### 2.11 Statistical analysis

The results were presented as the mean ± SD of at least three independent experiments. The Student's *t‐*test was used to assess the significance of the experimental and control groups. *p*‐value of <0.05 was considered statistically significant. SPSS version 24.0 was used to perform all statistical analyses.

## 3 RESULTS

### 3.1 Identification of overlapping DEGs in DCIS

**FIGURE 1:**
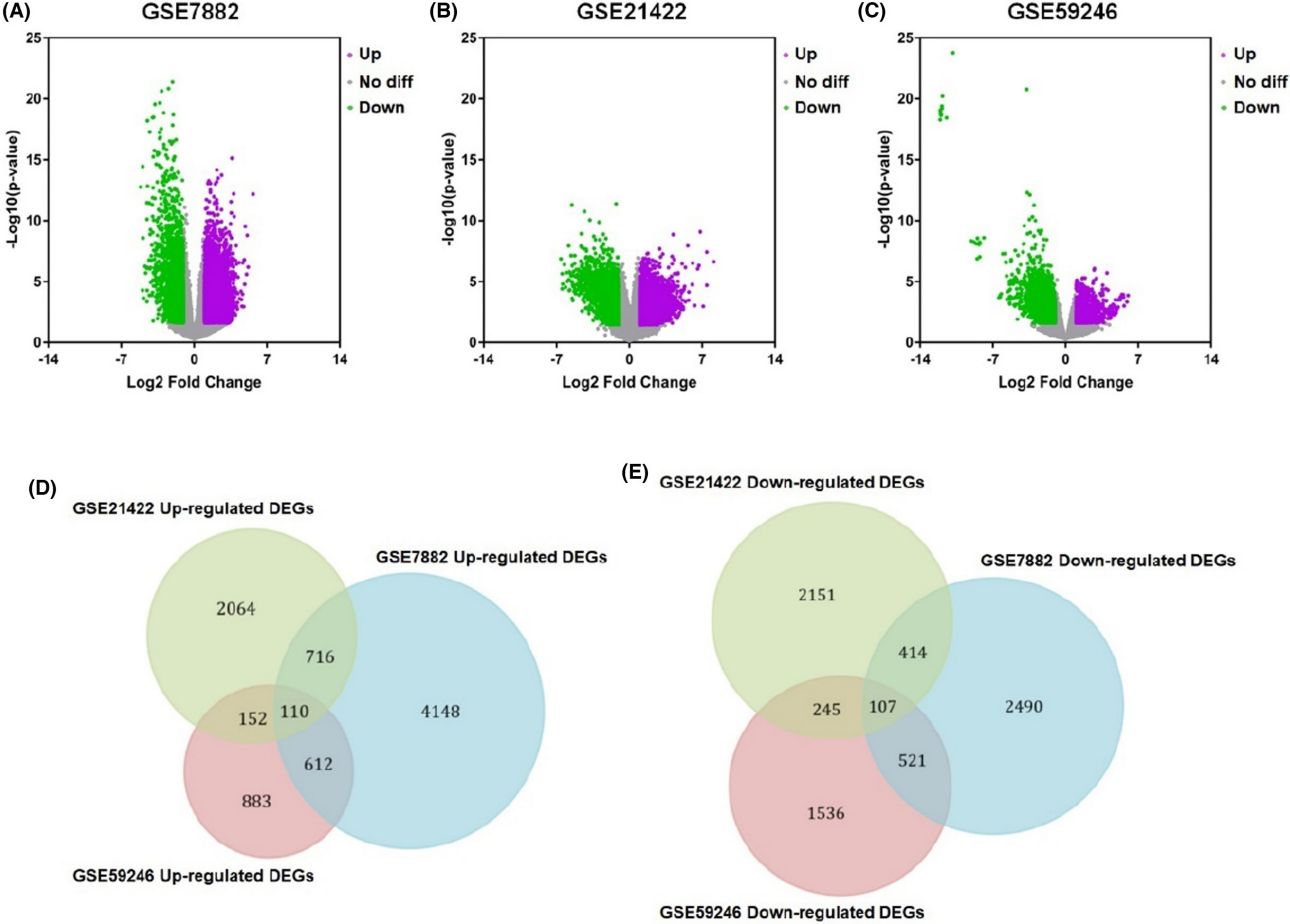
Identification of the differentially expressed genes in DCIS. (A–C) Upregulated and downregulated genes are represented by purple and green points. In gray points, there are no genes with differential expression. (A) DEGs based on GSE7882. (B) DEGs based on GSE21422. (C) DEGs based on GSE59246. (D) Co‐upregulated genes from GSE7882, GSE21422, and GSE59246 microarray profile datasets. (E) Co‐downregulated genes from GSE7882, GSE21422, and GSE59246 microarray profile datasets.

According to the cutoff criteria of *p* < 0.01 and |logFC| > 1 for selecting DEGs, a total of 5586, 3042, 1757 DEGs were recognized as upregulated, and 3532, 2917, 2409 DEGs were observed as downregulated from GSE7882, GSE21422, and GSE59246 microarray profile datasets (Figure 1A‐C), respectively. Figure 1D and E demonstrate that 110 genes were upregulated and 107 downregulated across the three datasets. The names of the overlapping genes are shown in Table 1.

**TABLE 1:** From three profile datasets, 217 differentially expressed genes were discovered, including 110 co‐upregulated genes and 107 co‐downregulated genes in DCIS

DEGs	Genes name
Upregulated	ATP2A2, POLE2, MELK, MAPKAPK2, P4HA3, BCOR, NEK2, TRIB3, VCAN, CXCL10, PDE7A, DHCR7, HOOK1, SLC20A1, TNC, PLEK2, B3GNT2, BDH1, CTHRC1, PTPN3, HNRNPH1, EFNA3, PAICS, CXADR, EPCAM, AFG3L2, SNRNP40, MTA1, PRCP, P4HA1, CDKN3, PHTF1, GTF3C2, ABI2, PABPC1, NUP210, STIP1, UBE2A, DEPDC1, POLR2H, CDH2, PAX5, YAF2, HNRNPA2B1, CUTA, MAPK8, BIRC5, IDH2, SCO2, DGKD, EMP2, CALU, DTL, PPAT, RPN2, CD44, NOP2, GALNT3, NRAS, TJP3, RFX5, CKAP4, GSS, CCT5, COL1A1, DDX31, ULK3, FBXO28, SLC45A4, TRA2B, KYNU, ACSL3, GPD2, TFDP1, RAB11A, SFPQ, TMPO, FGFR2, MYB, CREM, BCL2L11, SFI1, SLC35A2, BID, SPDL1, MTDH, EFS, ADAM12, COL1A2, MDM2, CYFIP2, REL, ACVR1B, GAPDH, TNFSF4, NUDCD1, ICA1, SRPK1, ZSCAN12, AREG, CUX1, AREL1, RIT1, PAIP1, RNF4, TFEC, SLC35D1, IFT80, AGR2, LMNB2
Downregulated	PCM1, CA3, SNX6, LIMCH1, TSC22D3, SNX2, PEPD, MYLK, HDAC7, HYAL1, RAP1B, KLF4, COL4A1, ARID4A, FOS, PALM, PHF11, ACKR3, TMEM218, KL, DNAJC18, MITF, MTHFD1, FYN, CD14, ADCY6, LYVE1, IL15RA, CTNNA1, TMEM255A, FOLR2, FKBP5, PTPN11, DCUN1D4, SLC40A1, LAMA3, RAB30, CDH13, STAB1, ING4, SCN4A, IFFO1, PDXK, DCN, MECOM, WDR45, SGK2, DHX38, HBB, DHDDS, KRIT1, EPHA3, ME3, EHBP1, SGCB, DGKZ, SEC22A, RUNX1T1, UGP2, PRPF31, FOSB, SCN9A, MAP1A, NCOR1, HNMT, ACSS2, ATXN3, RBP4, OGG1, CASP4, TEAD1, SORBS3, MEF2C, DENND3, ASMTL, ZHX1, SNTB2, CDON, CDIP1, ACTR3, SCUBE2, RPS6KA2, DYNC1I2, NFU1, CLU, MYO1C, ST3GAL6, ITGB5, CLDND1, OXTR, LY6K, TMCC1, RASSF8, ATP1A2, HEXA, USP24, SLC41A3, EXOC3, AADAC, PPBP, CYTH3, SNCA, HSD17B14, USP47, COLGALT2, IK, PCDH19

### 3.2 Functional enrichment analysis of overlapped DEGs in DCIS

**FIGURE 2:**
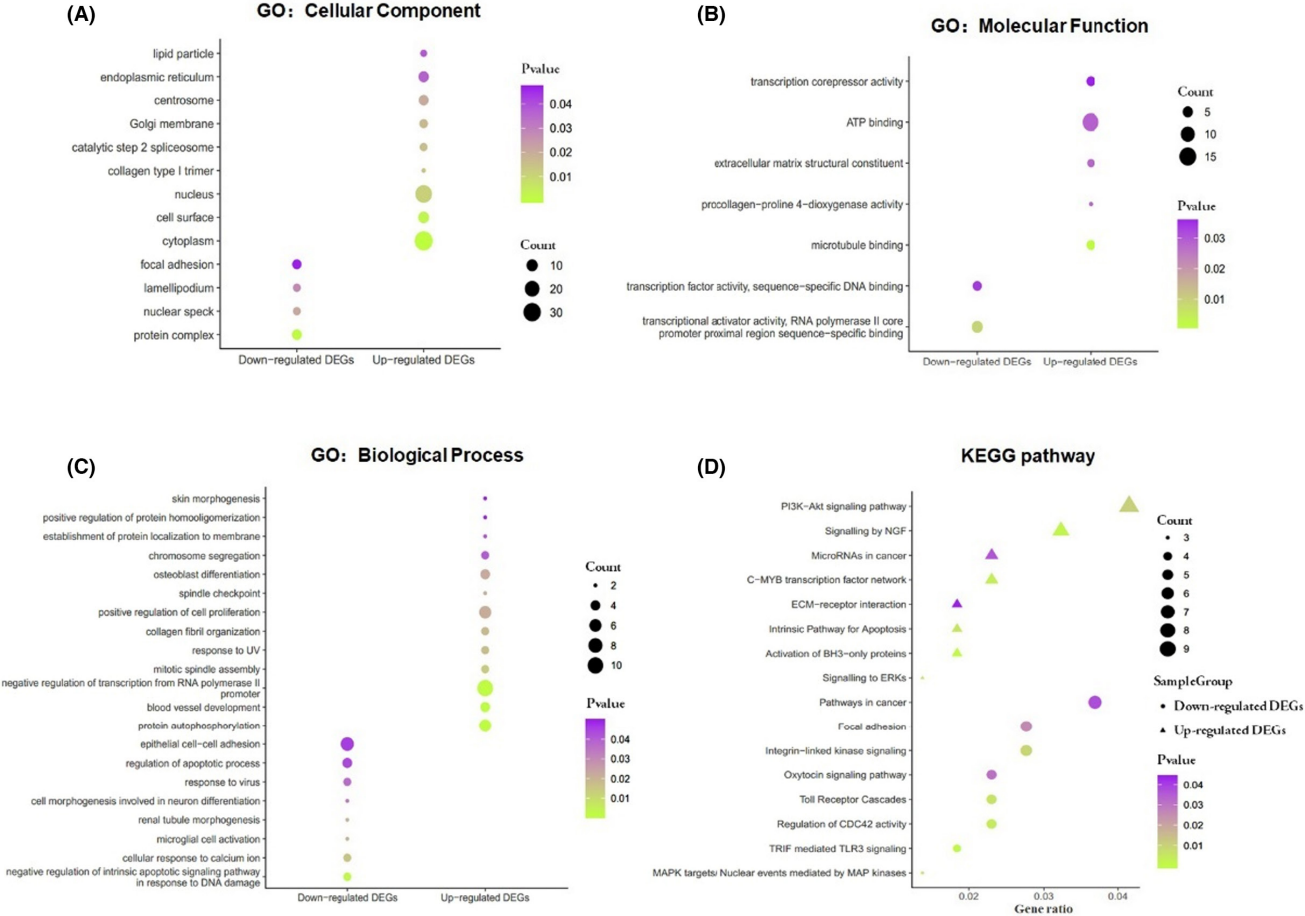
Analysis for signaling pathway and biology function of DEGs in DCIS. GO enrichment of (A) CC. (B) MF. (C) BP. (D) KEGG enrichment of signaling pathway.

GO‐enriched functions for the 217 overlapped DEGs were involved in various cellular components (CC), including cytoplasm, cell surface, nucleus, collagen type I trimer, and catalytic step 2 spliceosome for the upregulated genes, and protein complex, nuclear speck, lamellipodium, and focal adhesion for the downregulated genes (Figure 2A and Table 2). Microtubule binding, procollagen‐proline 4‐dioxygenase activity, extracellular matrix structural constituent, ATP binding, and transcription corepressor activity were included in the upregulated DEGs in molecular function (MF). In contrast, transcriptional activator activity, transcription factor activity, and RNA polymerase II core promoter proximal region sequence‐specific binding were included in the downregulated DEGs (Figure 2B and Table 2). For the biological processes (BP), the DEGs (upregulated) were enriched for protein autophosphorylation, blood vessel development, RNA polymerase II promoter‐based negative transcription regulation, mitotic spindle assembly, and response to UV. On the other hand, negative regulation in response to DNA damage of the inherent apoptotic signaling pathway, cellular response to calcium ion, renal tubule morphogenesis, microglial cell activation, and cell morphogenesis involved in neuron differentiation were all found to be downregulated DEGs (Figure 2C and Table 2).

**TABLE 2:** Gene ontology enrichment analysis of DEGs in DCIS

DEGs	Category	Term	Count	Fold enrichment	*p*‐value	FDR
Upregulated	GOTERM_BP_DIRECT	GO:0046777 ~ protein autophosphorylation	6	7.47	0.001	1.73
	GOTERM_BP_DIRECT	GO:0001568 ~ blood vessel development	4	18.25	0.001	1.88
	GOTERM_BP_DIRECT	GO:0000122 ~ negative regulation of transcription from RNA polymerase II promoter	10	3.63	0.001	2.29
	GOTERM_BP_DIRECT	GO:0090307 ~ mitotic spindle assembly	3	16.42	0.013	18.66
	GOTERM_BP_DIRECT	GO:0009411 ~ response to UV	3	14.66	0.017	22.67
	GOTERM_CC_DIRECT	GO:0005737 ~ cytoplasm	33	1.81	4.79E‐04	0.55
	GOTERM_CC_DIRECT	GO:0009986 ~ cell surface	9	3.81	0.002	2.70
	GOTERM_CC_DIRECT	GO:0005634 ~ nucleus	27	1.60	0.011	12.54
	GOTERM_CC_DIRECT	GO:0005584 ~ collagen type I trimer	2	14.05	0.014	15.17
	GOTERM_CC_DIRECT	GO:0071013 ~ catalytic step 2 spliceosome	4	7.49	0.015	16.98
	GOTERM_MF_DIRECT	GO:0008017 ~ microtubule binding	4	16.96	0.001	1.84
	GOTERM_MF_DIRECT	GO:0004656 ~ procollagen‐proline 4‐dioxygenase activity	2	19.98	0.027	28.32
	GOTERM_MF_DIRECT	GO:0005201 ~ extracellular matrix structural constituent	3	11.34	0.027	28.32
	GOTERM_MF_DIRECT	GO:0005524 ~ ATP binding	15	1.83	0.028	29.04
	GOTERM_MF_DIRECT	GO:0003714 ~ transcription corepressor activity	4	5.43	0.036	35.17
Downregulated	GOTERM_BP_DIRECT	GO:1902230 ~ negative regulation of intrinsic apoptotic signaling pathway in response to DNA damage	3	39.07	0.002	3.59
	GOTERM_BP_DIRECT	GO:0071277 ~ cellular response to calcium ion	3	15.35	0.015	20.92
	GOTERM_BP_DIRECT	GO:0061333 ~ renal tubule morphogenesis	2	95.51	0.020	26.33
	GOTERM_BP_DIRECT	GO:0001774 ~ microglial cell activation	2	95.51	0.020	26.33
	GOTERM_BP_DIRECT	GO:0048667 ~ cell morphogenesis involved in neuron differentiation	2	57.30	0.034	39.92
	GOTERM_CC_DIRECT	GO:0043234 ~ protein complex	7	7.00	4.65E‐04	0.54
	GOTERM_CC_DIRECT	GO:0016607 ~ nuclear speck	4	6.73	0.021	22.01
	GOTERM_CC_DIRECT	GO:0030027 ~ lamellipodium	4	6.04	0.027	28.09
	GOTERM_CC_DIRECT	GO:0005925 ~ focal adhesion	6	3.03	0.046	42.62
	GOTERM_MF_DIRECT	GO:0001077 ~ transcriptional activator activity, RNA polymerase II core promoter proximal region sequence‐specific binding	6	4.62	0.009	10.36
	GOTERM_MF_DIRECT	GO:0003700 ~ transcription factor activity, sequence‐specific DNA binding	4	5.57	0.034	33.91

Abbreviations: BP, Biological Process; CC, Cellular Component; Count, the number of enriched genes in each term; FDR, False Discovery Rate; MF, Molecular Function.

Furthermore, the DEGs' signaling pathways were enriched. The upregulated genes were associated with increased BH3‐only protein activation, NGF signaling, the C‐MYB transcription factor network, the intrinsic pathway for apoptosis, and ERK signaling. The downregulated genes were primarily involved in TRIF‐mediated TLR3 signaling, CDC42 activity regulation, MAPK targets/nuclear events mediated by MAP kinases, Toll receptor cascades, and integrin‐linked kinase signaling (Figure 2D and Table 3).

**TABLE 3:** Signaling pathway enrichment analysis of DEGs in DCIS

DEGs	Biological pathway	Count	*p*‐value	Mapped gene names
Upregulated	Activation of BH3‐only proteins	4	4.16E‐05	MAPK8, TFDP1, BCL2L11, BID
	Signaling by NGF	7	0.001	MAPKAPK2, TRIB3, MAPK8, NRAS, BCL2L11, MDM2, RIT1
	C‐MYB transcription factor network	5	0.003	PAX5, NRAS, MYB, COL1A2, TFEC
	Intrinsic pathway for apoptosis	4	0.004	MAPK8, TFDP1, BCL2L11, BID
	Signaling to ERKs	3	0.005	MAPKAPK2, NRAS, RIT1
	PI3K‐Akt signaling pathway	9	0.01	FGFR2, NRAS, TNC, EFNA3, COL1A2, MDM2, COL1A1, MYB, BCL2L11
	MicroRNAs in cancer	5	0.03	NRAS, CD44, TNC, MDM2, BCL2L11
	ECM‐receptor interaction	4	0.04	CD44, TNC, COL1A2, COL1A1
Downregulated	TRIF‐mediated TLR3 signaling	4	0.000	FOS, PTPN11, MEF2C, RPS6KA2
	Regulation of CDC42 activity	5	0.003	HDAC7, KLF4, FOS, MITF, FYN
	MAPK targets/ nuclear events mediated by MAP kinases	3	0.005	FOS, MEF2C, RPS6KA2
	Toll receptor cascades	5	0.005	FOS, CD14, PTPN11, MEF2C, RPS6KA2
	Integrin‐linked kinase signaling	6	0.009	HDAC7, KLF4, FOS, MITF, FYN, FKBP5
	Focal adhesion	6	0.025	COL4A1, LAMA3, FYN, ITGB5, RAP1B, MYLK
	Oxytocin signaling pathway	5	0.031	MEF2C, FOS, ADCY6, OXTR, MYLK
	Pathways in cancer	8	0.037	FOS, COL4A1, LAMA3, MITF, ADCY6, RUNX1T1, MECOM, CTNNA1

*Note*: Count, the number of enriched genes in each term.

### 3.3 Module analysis and PPI network construction

**FIGURE 3:**
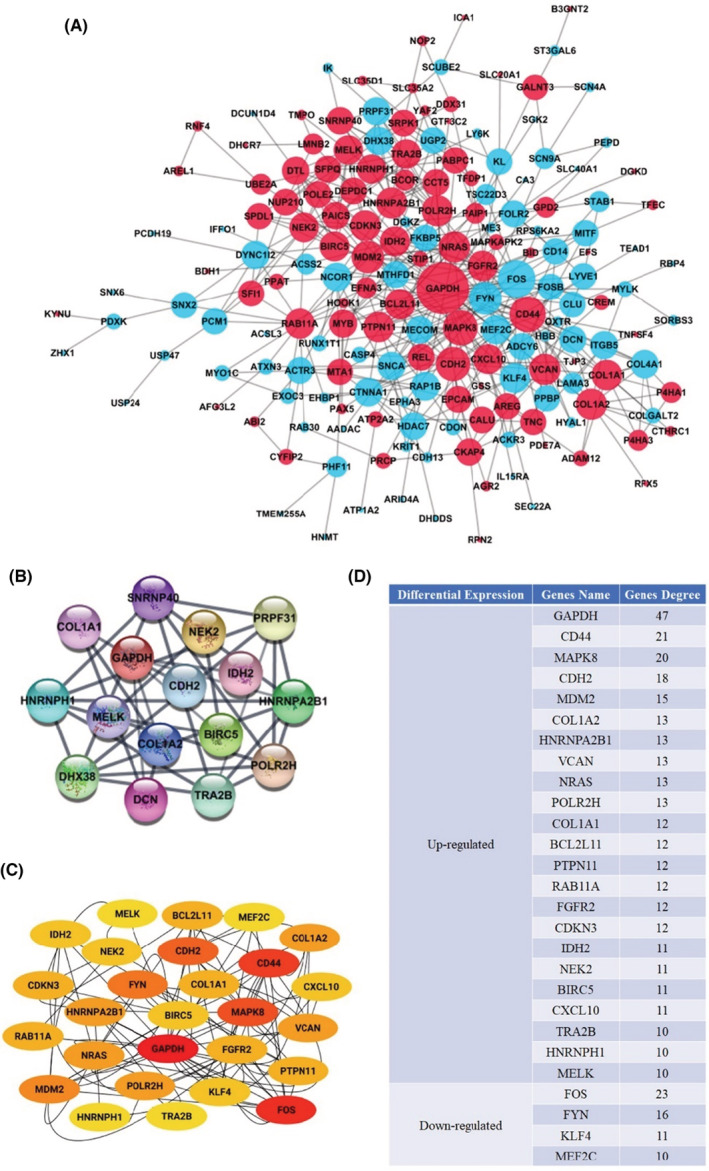
Module analysis and DEGs PPI network complex. (A) STRING and Cytoscape analysis‐based DEG PPI network. The red nodes depict upregulated genes, the light blue nodes represent downregulated genes, and the lines represent the DEGs' interactions. (B) The Cytoscape MCODE plugin‐based identification of the most significant module was performed. (C) The first 27 genes were chosen using the CytoHubba plugin. The redder color represents the more front‐ranking. (D) Genes' name and high degree node values. The amount of additional nodes connected to a node is defined as a node degree.

The DEG overlapping revealed a unique set of networks and interactions. The online database of STRING was used to filter 178 DEGs (92 upregulated and 86 downregulated genes) from the 217 usually altered DEGs belonging to the PPI network complex. A total of 39 DEGs were excluded from the PPI network. Furthermore, using Cytoscape software analysis, 456 edges were identified in overlapping DEGs. The degree of the PPI network complex defaulter filter ranged from 1 to 64 (Figure 3A).

Furthermore, the entire PPI network was analyzed through Cytoscape's MCODE plugin; the most significant module and 16 nodes were identified using degree cutoff = 2, k‐core = 2, node score cutoff = 0.2, and maximum depth = 100 as the criterion (Figure 3B). Afterward, as shown in Figure 3C and Figure 3D, the first 27 PPI network genes were chosen using the CytoHubba plugin, and node degrees were analyzed. Twelve core candidate genes were selected after combining the results of MCODE, CytoHubba, and nodes degree, and all of them were upregulated DEGs, in the following order: GAPDH, CDH2, COL1A2, HNRNPA2B1, POLR2H, COL1A1, IDH2, NEK2, BIRC5, TRA2B, HNRNPH1, and MELK. They may significantly impact the progression of DCIS and the prognosis.

### 3.4 Kaplan–Meier survival analysis

**FIGURE 4:**
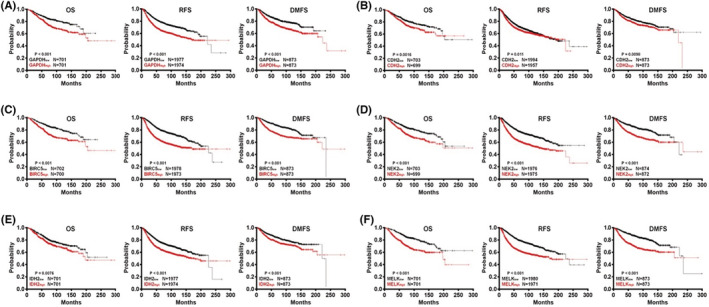
Prognostic of core candidate genes in DCIS according to the Kaplan–Meier plotter database. (A) GAPDH (M33197_3_at). (B) CDH2 (203440_at). (C) BIRC5 (202095_s_at). (D) NEK2 (204641_at). (E) IDH2 (210046_s_at). (F) MELK (204825_at). Patients with high gene expression are represented by red lines, whereas black lines exemplify those with low gene expression. OS stands for overall survival. RFS stands for “relapse‐free survival.” Distance metastasis‐free survival is abbreviated as DMFS.

The Kaplan–Meier survival plot was utilized to evaluate the prognostic details of the 12 core candidate genes. The 12 genes, including GAPDH, CDH2, COL1A2, HNRNPA2B1, POLR2H, COL1A1, IDH2, NEK2, BIRC5, TRA2B, HNRNPH1, and MELK, were used to plot the survival curves by uploading them to database. High expression of GAPDH (*p* = 7.9e‐07), CDH2 (*p* = 0.0016), BIRC5 (*p* = 1.6e‐08), NEK2 (*p* = 1.3e‐06), IDH2 (*p* = 0.0076), and MELK (*p* = 9.2e‐11) were correlated with poor patient's OS, while COL1A2 (*p* = 0.53), HNRNPA2B1 (*p* = 0.16), POLR2H (*p* = 0.051), COL1A1 (*p* = 0.28), TRA2B (*p* = 0.55), and HNRNPH1 (*p* = 0.46) expressions were not relevant to OS (Figure 4 and Figure S1). Survival curves also showed that high expression of GAPDH (*p* = < 1e‐16 and 2.9e‐06), CDH2 (*p* = 0.011 and 0.0098), BIRC5 (*p* = <1e‐16 and 6e‐10), NEK2 (*p* = <1e‐16 and 5.3e‐10), IDH2 (*p* = 7.8e‐14 and 0.00049), and MELK (*p* = <1e‐16 and 4.9e‐14) were significantly associated with worse RFS and DMFS (Figure 4).

### 3.5 Validation of prognostic effectiveness of the hub genes

**FIGURE 5:**
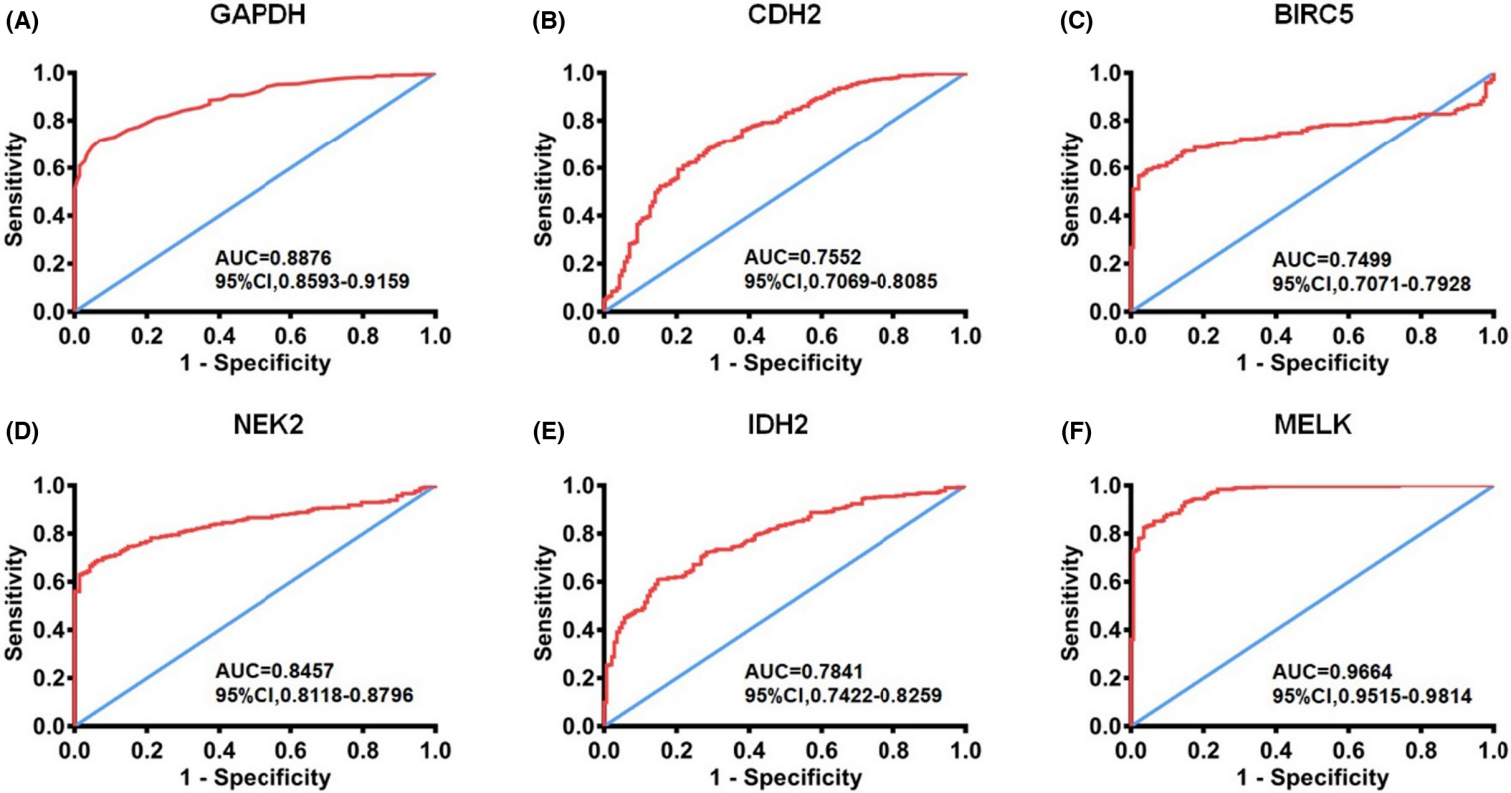
Validation ROC curves of hub genes based on TCGA cohort. (A) ROC curve of GAPDH. (B) ROC curves of CDH2. (C) ROC curves of BIRC5. (D) ROC curves of NEK2. (E) ROC curves of IDH2. (F) ROC curves of MELK. Red represents sensitive curves; blue indicates identity lines. ROC, receiver operating characteristic; AUC, Area under the curve; CI, Confidence interval.

Based on TCGA samples, the expression of the selected six hub genes with prognostic significance was further investigated. The six upregulated hub genes were subjected to ROC analysis, which shows a favorable prognostic value for DCIS. Moreover, the area under curve (AUC) of GAPDH, CDH2, BIRC5, NEK2, IDH2, and MELK was 0.8876, 0.7552, 0.7499, 0.8457, 0.7841, and 0.9664 (Figure 5A–F), respectively.

### 3.6 Prognostic analysis and core gene signatures

**FIGURE 6:**
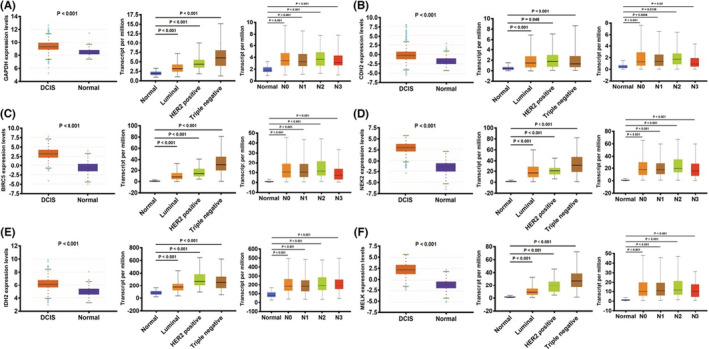
Core gene expression overview according to the TCGA samples. The candidate gene's mRNA expression differences, based on sample types, molecular subtypes, and lymph node metastasis status. (A) GAPDH. (B) CDH2. (C) BIRC5. (D) NEK2. (E) IDH2. (F) MELK.

DCIS has been linked to six core genes. The increased expression of core genes in DCIS samples has been illuminated. There were also differences in the upregulated degree of core genes in diverse breast cancer molecular types. The expression of GAPDH, NEK2, BIRC5, and MELK was higher in triple‐negative breast cancer, a subtype with poor prognoses, and CDH2 and IDH2 were higher in HER2‐positive subtype. The upregulated degree was higher in the subtype with a poor prognosis. Additionally, high expression of core genes also increases the risk of lymph node metastasis. However, it is not that the higher expression of core genes, the later of the *N* stage. (Figure 6A–F).

### 3.7 Downregulation of core gene expression inhibits the proliferation

**FIGURE 7:**
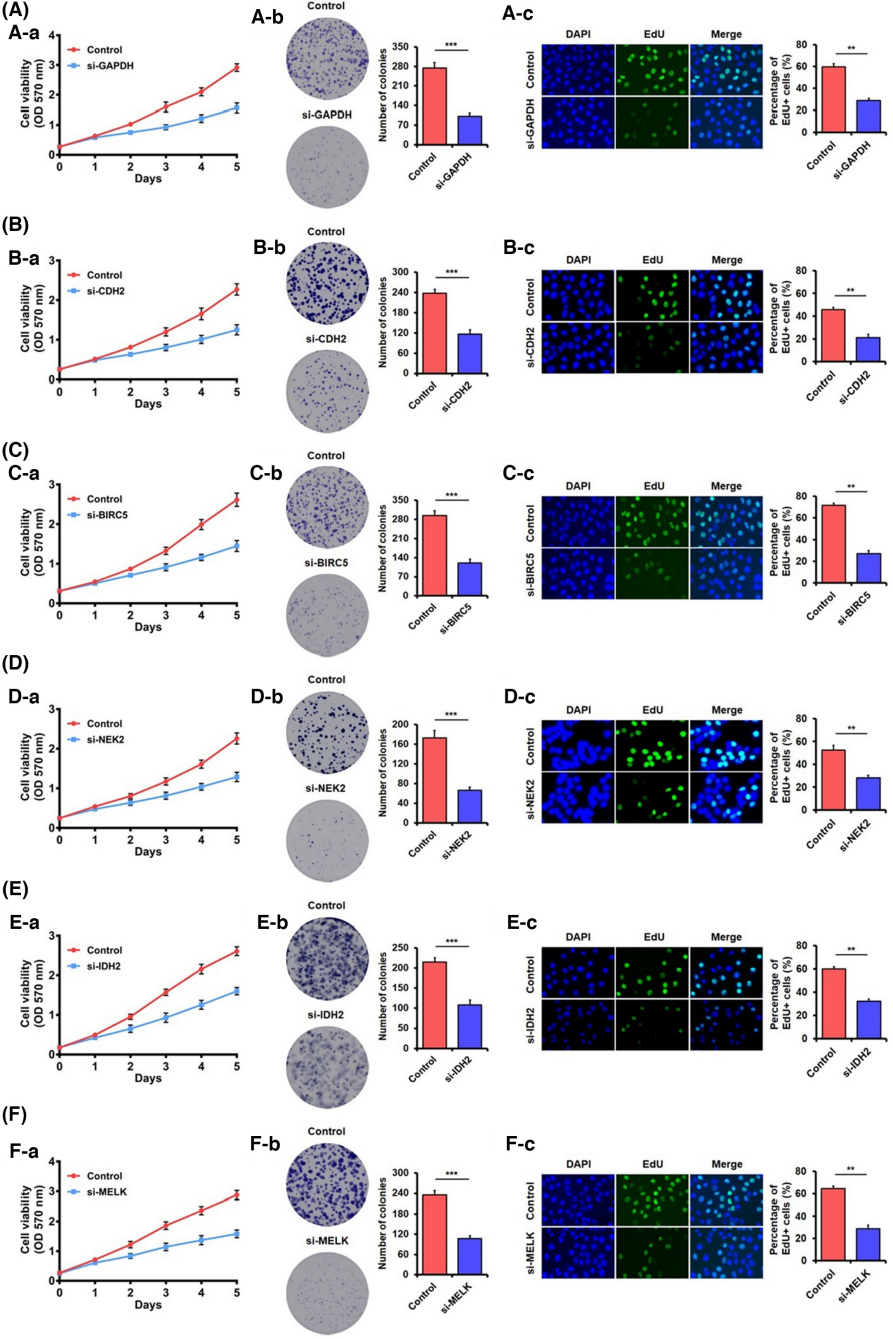
Knockdown of core genes inhibits cell proliferation demonstrated through MTT assay, colony formation assay, EdU assay. (A) Down expression of GADPH inhibits MDA‐MB‐231 cell growth. (B) Down expression of CDH2 inhibits T47D cell growth. (C) Down expression of BIRC5 inhibits MDA‐MB‐231 cell growth. (D) Down expression of NEK2 inhibits MCF‐7 cell growth. (E) Down expression of IDH2 inhibits SK‐BR‐3 cells growth. (F) Down expression of MELK inhibits MDA‐MB‐231 cell growth. The data were shown as mean ± SD obtained from at least three independent experiments. The statistical significance (**, *p* < 0.01; ***, *p* < 0.001) is determined through Student's *t‐*test. (a) MTT assay, (b) colony formation assay, (c) EdU assay.

T47D, MDA‐MB‐231, SK‐BR‐3, MCF‐7, BT474, and BT549 are common breast cancer cells. MCF10A is the normal mammary epithelial cell. RT‐qPCR was used to compare the core gene expressions in breast cancer and normal cell lines. The result shows that GAPDH, BIRC5, and MELK expressions were elevated in MDA‐MB‐231, CDH2 was highly expressed in T47D, NEK2 in MCF‐7, and IDH2 was highly expressed in SK‐BR‐3 (Figure S2). Subsequently, transfection with siRNAs for the downregulation of core gene expression in the corresponding cells (Figure S3). The cell proliferation of breast cancer was inhibited when the expression of core genes was downregulated through MTT, colony formation, and EdU assays (Figure 7A–F). Data to support using a six‐gene signature for DCIS diagnosis and prognosis prediction include GAPDH, CDH2, BIRC5, NEK2, IDH2, and MELK.

## 4 DISCUSSION

DCIS is a heterogeneous disease that defines the stage of breast cancer before it becomes invasive.^17^ While most DCIS patients have excellent long‐term results, some DCIS patients can still develop invasive breast cancer. Unfortunately, current clinical methods result in overtreatment of certain women with DCIS due to confusion about which lesions are at risk of progressing to invasive cancer. As a result, the identification of novel prognostic biomarkers is critical. Gene signatures based on aberrant mRNAs have shown considerable potential in predicting cancer prognosis.

In this study, we investigated the gene expression profiles of 148 DCIS patients and discovered 217 common DEGs, including 101 upregulated and 138 downregulated genes. According to the functional enrichment results, the DEGs mainly were associated with protein autophosphorylation, cytoplasm, microtubule binding, negative regulation of intrinsic apoptotic signaling pathway in response to DNA damage, protein complex, transcriptional activator activity, and RNA polymerase II core promoter proximal region sequence‐specific binding. Signaling pathway enrichment analysis is associated with activating BH3‐only proteins, and TRIF mediated TLR3 signaling. With the help of the PPI network, 12 hub genes were selected for further analysis. While the GAPDH, CDH2, BIRC5, NEK2, IDH2, and MELK were the negative prognostic genes in DCIS patients. ROC and signatures analysis demonstrated that the core genes could be a valuable indicator for DCIS. Additionally, downregulation of core gene expression through small interfering RNAs transfection inhibits the proliferation of breast cancer cells significantly, suggesting a great potential for utilizing the core genes in DCIS prognosis.

GAPDH, or glyceraldehyde‐3‐phosphate dehydrogenase, is a housekeeping gene that often serves as an internal control in experiments. Increased GAPDH levels, on the other hand, are seen in a range of human cancers and are often linked to shorter survival times.^18^, ^19^, ^20^, ^21^ The evidence suggests that GAPDH function mechanisms, such as its role in cell survival of tumor, angiogenesis, and posttranscriptional regulation of tumor cell mRNA, are associated with poor prognosis and increased tumor progression for the affected individual.^22^, ^23^

Surprisingly, the role and mechanism of increased GAPDH in DCIS remain unknown. CDH2 (Cadherin 2), as a member of the cadherin superfamily, encodes the N‐cadherin protein, which plays an imperative role in EMT (epithelial‐mesenchymal transition). Elevated expression of CDH2 implicated poor prognosis in various cancers such as lung cancer,^24^ prostate cancer,^25^ ^,^ and glioma.^26^ Primarily, CDH2 was found to be overexpressed in DCIS with invasion, which may be an early marker in the absence of histological signs and a marker of a short‐term local recurrence after treatment.^27^ BIRC5 (also known as Survivin) is an apoptosis inhibitory protein that exerts a role in inhibiting cell death and promoting cancer cell survival.^28^ Studies showed that BIRC5 expression is significantly increased in lung, breast, and colon cancers.^29^, ^30^ BIRC5 can be used as a predictor marker in different tumors due to its aggregation. As a result, increased survivin expression could be regarded as a prognostic marker associated with increased lymph node invasion, recurrence possibility, and metastasis.^31^, ^32^

NEK2, never‐in‐mitosis (NIMA)‐related kinase 2, plays a crucial role in regulating microtubule stabilization, centrosome separation and duplication, spindle assembly checkpoint, and kinetochore attachment.^33^ Evidence suggests that the level of NEK2 is upregulated in primary tumor tissues or cancer cell lines.^34^, ^35^, ^36^ Furthermore, increased NEK2 overexpression is linked with advanced tumor stage, distant metastases, and lymph node invasion, suggesting that it may be used to predict tumor progression and disease prognosis.^37^, ^38^, ^39^ IDH2, isocitrate dehydrogenase 2, performs the oxidative decarboxylation of isocitrate to α‐ketoglutarate (α‐KG). IDH2 is the most commonly mutated metabolic gene in cancer, and it disrupts metabolic and epigenetic regulation, promoting tumorigenesis in humans.^40^

Interestingly, the IDH2 frequently showed overexpression rather than a mutation in the bladder, breast, and lung cancers. According to Li et al., upregulated wild‐type IDH2 promotes proliferation and tumor formation in the lung cancer cell and is linked to a lower overall survival rate.^41^ IDH2 has been related to DCIS recurrence and progression to invasive disease and is expressed differently in recurrent and non‐recurrent DCIS. Furthermore, high wild‐type IDH2 expression was linked to a poor patient outcome in DCIS.^42^, ^43^, ^44^ According to the microarray and TCGA datasets study, MELK (maternal embryonic leucine zipper kinase) expression is higher in many cancer cells and tissues than their counterparts.^45^, ^46^, ^47^ MELK expression levels are also associated with high‐grade tumors, increased aggressiveness, and poor patient outcomes.^48^, ^49^, ^50^ According to research, MELK has been recognized as an efficient therapeutic target and prognostic factor in the treatment of cancer.

According to evidence in the literature, the core genes play a significant role in cancer progression through various mechanisms. GAPDH, for example, regulates the apoptosis signaling system to increase tumor cell survival by reducing H_2_O_2_‐induced programmed cell death and mediated suppression of caspase‐independent cell death.^23^, ^51^ CDH2 plays a vital role in the transition from epithelial to mesenchymal state (EMT) and allows abnormal cells to invade and proliferate to surrounding and distant tissues.^52^ As a cancer driver gene, IDH2 can promote tumor progression via interaction between histone demethylation and hypoxia reprogramming in cancer metabolism.^53^ Overall, our data indicate that the six core genes may be helpful as predictive and diagnostic biomarkers for DCIS. However, additional research is needed to determine these six genes' expression and prognostic function at the protein level. Therefore, functional experiments are required to elucidate their underlying mechanism.

## 5 CONCLUSIONS

It is essential to identify biomarkers with potential for DCIS diagnosis and prognosis prediction. While investigating genes as DCIS biomarkers, a critical element is identifying a panel of deregulated genes that can improve the biomarker's sensitivity and specificity rather than identifying individual genes. Our current findings validated the six core genes signature for potential DCIS biomarkers, which may facilitate clinical decision‐making for individual care.

## AUTHORS' CONTRIBUTIONS

Jiao Zhang: Conceptualization (Lead); Data curation (Lead); Formal analysis (Lead); Investigation (Lead); Methodology (Lead); Resources (Lead); Software (Lead); Writing‐original draft (Lead). Hui Lin: Data curation (Lead); Formal analysis (Lead); Investigation (Lead); Methodology (Lead); Resources (Lead); Software (Lead); Writing‐original draft (Lead). Lei Hou: Formal analysis (Equal); Methodology (Equal); Software (Equal). Hui Xiao: Formal analysis (Equal); Methodology (Equal); Software (Equal). Xilong Gong: Formal analysis (Equal); Methodology (Equal); Software (Equal). Xuhui Guo: Formal analysis (Equal); Methodology (Equal); Software (Equal). Xuchen Cao: Project administration (Lead); Supervision (Lead); Validation (Lead); Writing‐review & editing (Lead). Zhenzhen Liu: Project administration (Lead); Supervision (Lead); Validation (Lead); Writing‐review & editing (Lead).

## FUNDING INFORMATION

This research was funded by the Science and Technology Development Plan of Henan Province (priority development and promotion projects) (222102310147), the scientific and technological research projects of Henan Province (SBGJ202102065).

## CONFLICT OF INTEREST

The authors confirm that there are no conflicts of interest.

## CONSENT FOR PUBLICATION

All the authors have reviewed the manuscript and the related files and consented to its publication.

## Supporting information


Figures S1‐S3

Tables S1‐S2

## Data Availability

Data sharing is not applicable to this article as no new data were created or analyzed in this study.
