# Supplementary material for: Exploration of the breast ductal carcinoma in situ signature and its prognostic implications
Source: Cancer Med. 2022 Jul 26;12(3):3758–72. doi: 10.1002/cam4.5071 (PMC9939111; doi:10.1002/cam4.5071)
Supplement: Supplementary file 1 — Figures S1‐S3 [file CAM4-12-3758-s002.docx]

**Supplement Figures**


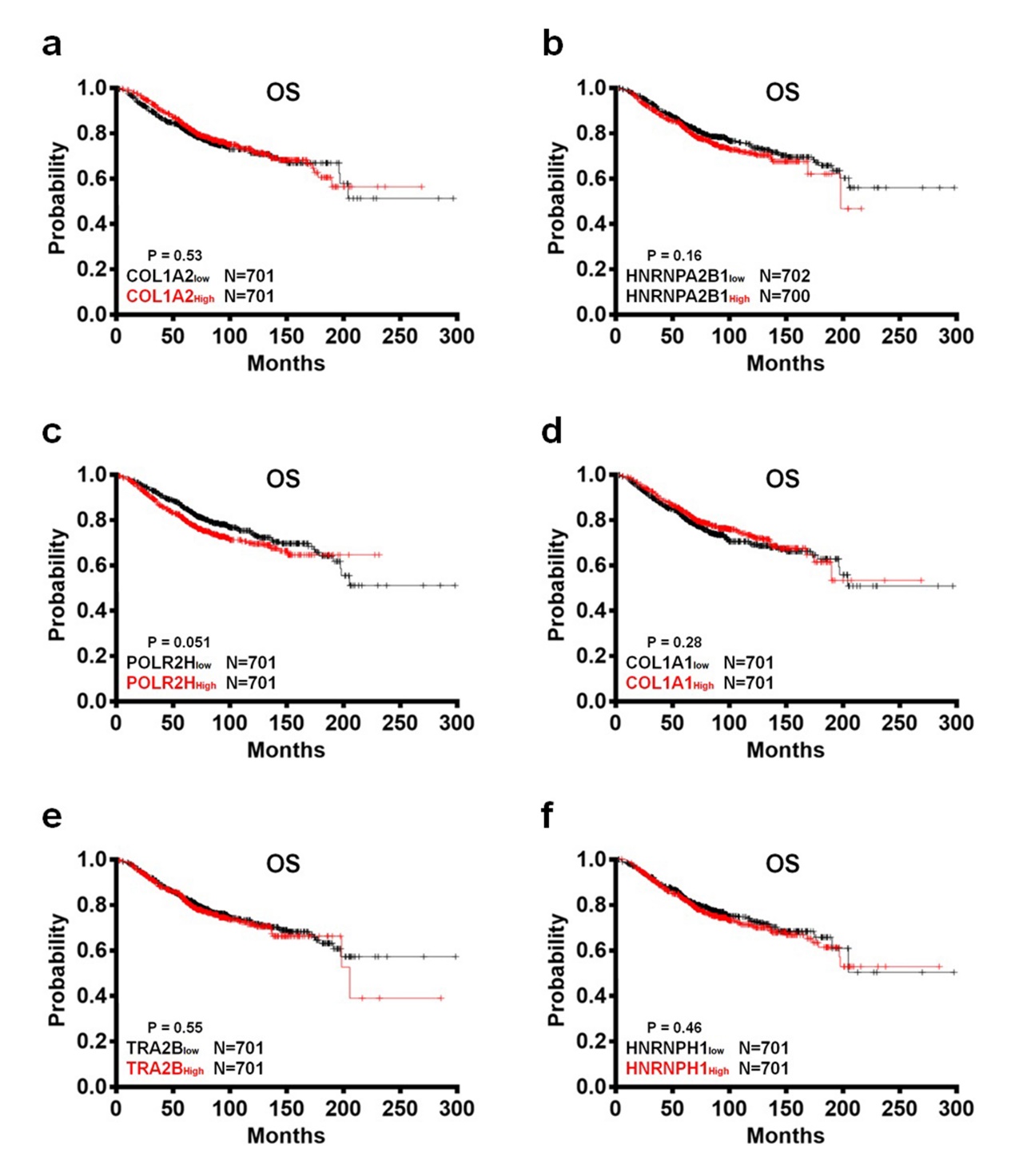


**FIGURE S1** Overall survival of hub candidate genes in DCIS according to the Kaplan–Meier plotter database. **a** COL1A2 (202404_s_at). **b** HNRNPA2B1 (205292_s_at). **c** POLR2H (209302_at). **d** COL1A1 (202311_s_at). **e** TRA2B (200892_s_at). **f** HNRNPH1 (213619_at). Patients with high gene expression are represented by red lines, whereas those with low gene expression are represented by black lines.


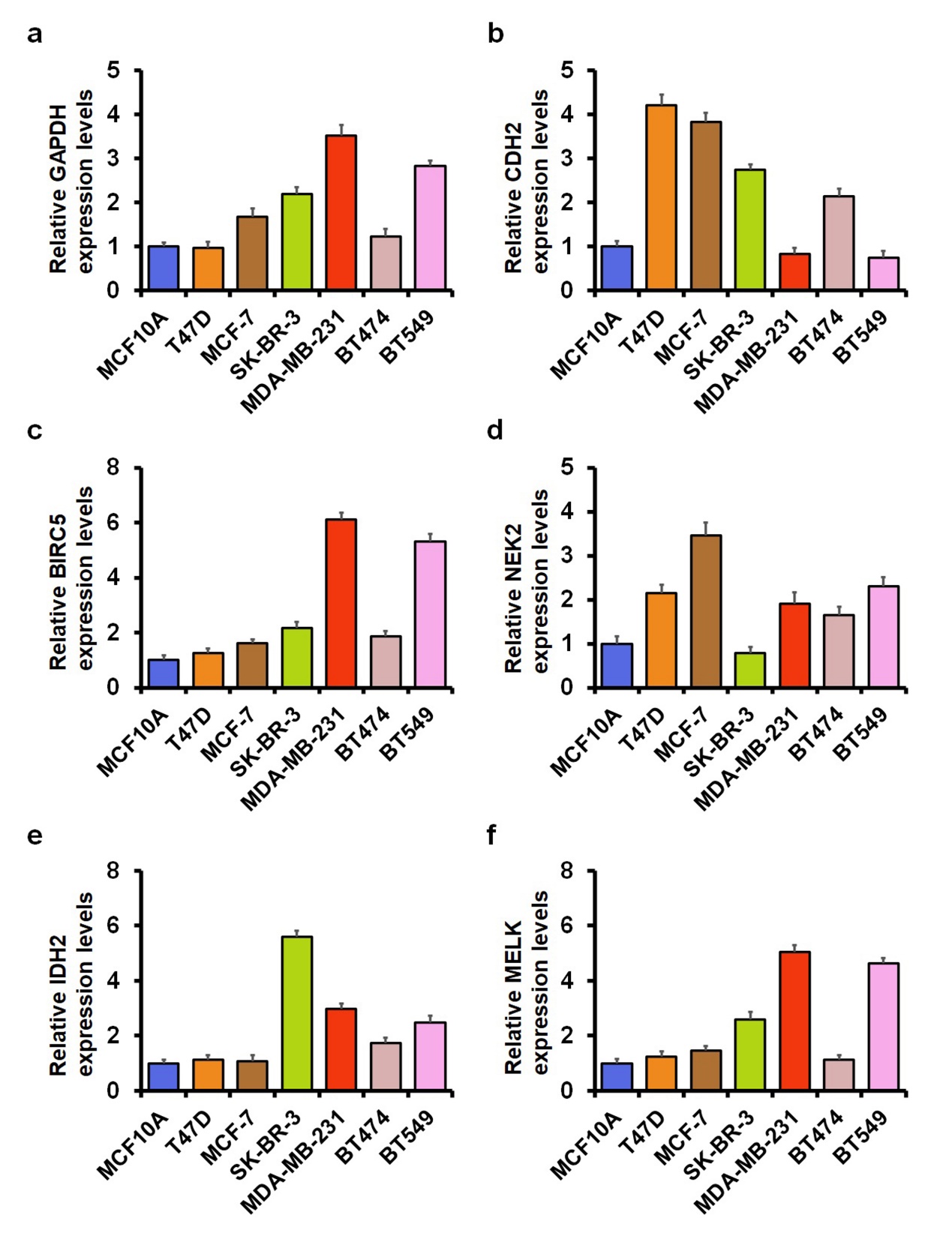


**FIGURE S2** RT-qPCR based assessment for the expression of core candidate genes in breast cancer cell lines. **a** GAPDH expression. **b** CDH2 expression. **c** BIRC5 expression. **d** NEK2 expression. **e** IDH2 expression. **f** MELK expression, in breast cancer cell lines.


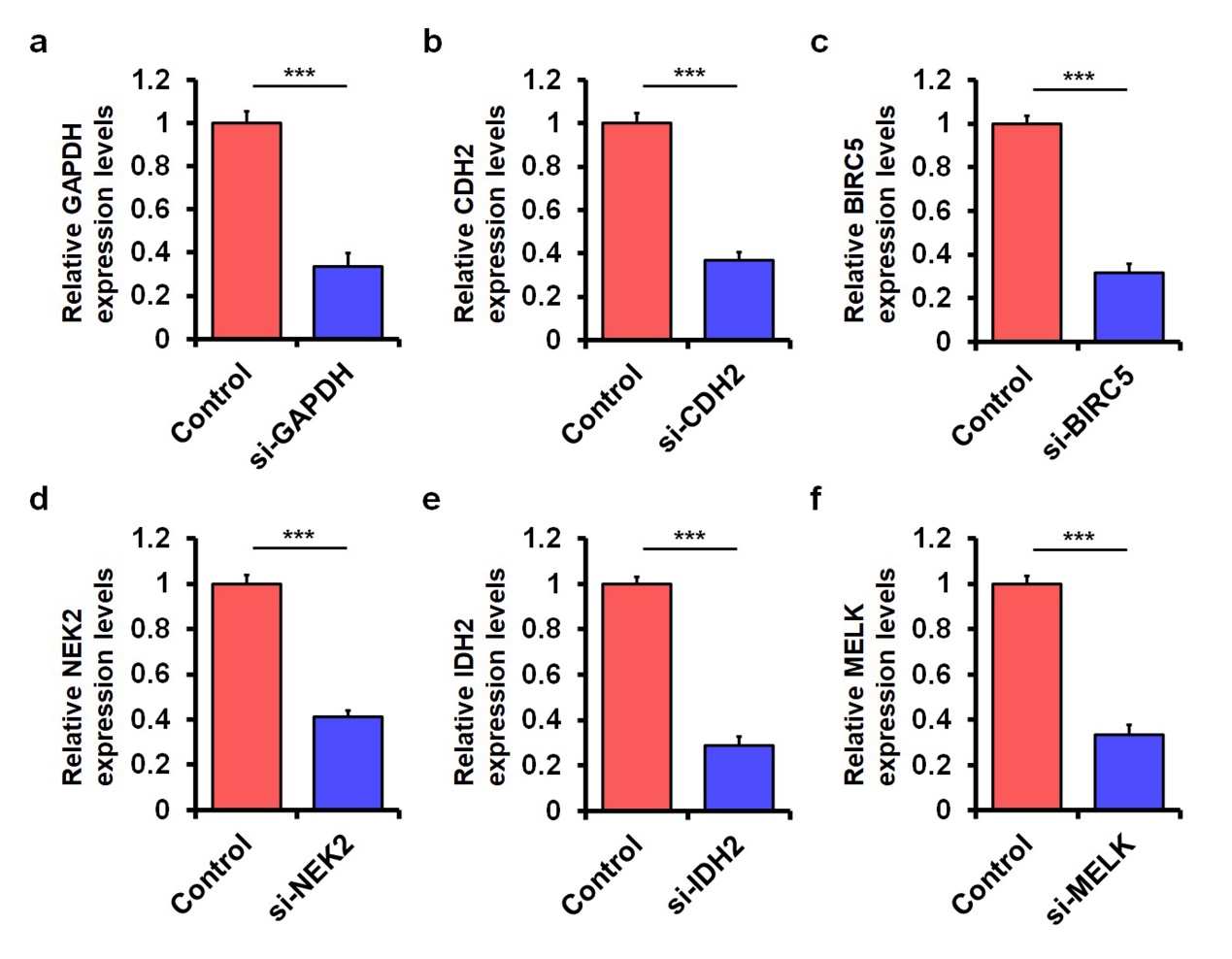


**FIGURE S3** RT-qPCR based assessment for the core genes expression in breast cancer cell transfected with small interfering RNAs. **a** GAPDH expression in MDA-MB-231 cells. **b** CDH2 expression in T47D cells. **c** BIRC5 expression in MDA-MB-231 cells. **d** NEK2 expression in MCF-7 cells. **e** IDH2 expression in SK-BR-3 cells. **f** MELK expression in MDA-MB-231 cells. The data were shown as mean ± SD obtained from at least three totally independent studies. The level of significance was determined by Student’s t-test. ***, p < 0.001.
