# Supplementary material for: Exploration of the breast ductal carcinoma in situ signature and its prognostic implications
Source: Cancer Med. 2022 Jul 26;12(3):3758–72. doi: 10.1002/cam4.5071 (PMC9939111; doi:10.1002/cam4.5071)
Supplement: Supplementary file 2 — Tables S1‐S2 [file CAM4-12-3758-s001.docx]

**Supplement table**

| **Genes** | **Primer sequence of RT-qPCR** |
| --- | --- |
| GAPDH | Forward: 5'-CCTTCATTGACCTCAACTA-3'  Reverse: 5'- GGAAGGCCATGCCAGTGAGC-3' |
| CDH2 | Forward: 5'-TGCGGTACAGTGTAACTGGG-3'  Reverse: 5'-GAAACCGGGCTATCTGCTCG-3' |
| BIRC5 | Forward: 5'-ACGACCCCATAGAGGAACAT-3'  Reverse: 5'-TCCGCAGTTTCCTCAAATTC-3' |
| NEK2 | Forward: 5'-TGCTTCGTGAACTGAAACATCC-3'  Reverse: 5'-CCAGAGTCAACTGAGTCATCACT-3' |
| IDH2 | Forward: 5'-CGCCACTATGCCGACAAAAG-3'  Reverse: 5'-ACTGCCAGATAATACGGGTCA-3' |
| MELK | Forward: 5'-CATTAGCCCTGAGAGGCGGTGC-3'  Reverse: 5'-GCCCGTCTCTGGCAGAACCCTT-3' |
| β-actin | Forward: 5'-CCTCGCCTTTGCCGATCC-3'  Reverse: 5'-GGATCTTCATGAGGTAGTCAGTC-3' |

**TABLE S1** Primer sequence of core genes RT-qPCR.

| **Genes** | Target sequence |
| --- | --- |
| GAPDH | AGGTCGGAGTCAACGGATT |
| CDH2 | CCAATCAACTTGCCAGAAA |
| BIRC5 | GGTTCCTTATCTGTCACAC |
| NEK2 | CATCGTTCGTTACTATGAT |
| IDH2 | ACGTGGACATCCAGCTAAA |
| MELK | CTGCCATATCCTTACTGGA |

**TABLE S2** Small interfering RNA target sequence to the core genes.
